# Claims in the clinic: A qualitative group interview study on healthcare communication about unestablished side effects of the copper IUD

**DOI:** 10.1371/journal.pone.0291966

**Published:** 2023-09-28

**Authors:** Maria Wemrell, Lena Gunnarsson

**Affiliations:** 1 Department of Social Work, Linnaeus University, Växjö, Sweden; 2 Unit for Social Epidemiology, Department of Clinical Sciences, Lund University, Malmö, Sweden; 3 School of Humanities, Education and Social Sciences, Örebro University, Örebro, Sweden; University of KwaZulu-Natal College of Health Sciences, SOUTH AFRICA

## Abstract

**Background:**

Lay online communication about health-related issues has in recent years largely been associated with the spread of misinformation and decreased trust in healthcare. Such communication has included claims about systemic side effects of the copper IUD. In Sweden, a social media group centered on this issue now gathers around 8,700 members. This study aimed to use the case of reported yet unestablished side effects of the copper IUD to investigate experiences of and reasoning about healthcare encounters between caregivers and patients contesting established medical knowledge.

**Methods:**

We conducted qualitative, semi-structured, digital group interviews with members of the social media group (seven groups, n = 23) and with midwives and gynecologists (six groups, n = 15). We also gathered essays written by social media group members (n = 23). The material was analyzed thematically.

**Results:**

The participant accounts pointed towards tensions related to principles of evidence-based medicine, i.e., perceived insufficiency of research on the safety of the copper IUD and lack of clarity in routines for reporting and following up suspected side effects, and of patient-centered care, i.e., listening respectfully to patients. Tension between caregivers’ obligation to adhere to evidence-based medicine while also providing patient-centered care was noted.

**Conclusion:**

Healthcare providers’ efforts to assess and address patient claims contesting established medical knowledge should include ensuring and communicating sufficient research, clarifying procedures for reporting suspected side effects, and improving person-centered care. This can increase the quality of care while contributing to the mitigation of distrust in healthcare and the spreading of health-related misinformation.

## Introduction

Over recent decades, the internet and particularly social media have created unprecedented opportunities for lay people to seek and share health-related information. A large share of women, in particular, use the internet to find and share such information [1]. In 2015, the internet was reportedly the primary source of information about contraceptives for two-thirds of young women in Sweden [2].

Lay online communication about health has been linked to increased contestation of expert knowledge [3] and the spread of misinformation [4, 5], associated with public knowledge deficits or misunderstanding of science [6, 7], potentially threatening individual and public health [1, 5]. This issue has been actualized during recent years, most intensly in relation to vaccine hesitancy [8–10] but also in connection to contraceptive methods [11–13].

At the same time, noted effects of seeking and sharing health information online include increased involvement in and knowledge about health [1, 14], potential alleviation of health inequalities [14] and democratization of information exchange among less privileged groups [15, 16]. Lay online engagement with medical knowledge can here be related to the historical background of health movements developing from the 1960s onwards, challenging medical authority not least in relation to reproductive and other women’s health issues [17, 18]. Such efforts have included attendance to side effects and coercive practices associated with contraceptives [19–22], on occasion contributing to litigation or product recall due to previously unknown risks [20, 21, 23]. Research has furthermore pointed to patients finding it difficult to express their concerns about side effects of contraceptives to, and receive sufficient information about them from, caregivers [24–26], and to healthcare providers downplaying or dismissing such side effects or women’s concerns about them [27–33], in countries including Sweden [34, 35]. This is while side effects of contraceptives for women have been deemed more tolerable than those of contraceptives for men, even as women suffered more severe health outcomes [36], and the embodied experiences of women have historically been taken as unreliable sources of knowledge in medical contexts [37].

Moreover, structures and practices in healthcare through which some persons or groups are construed as knowledgable and others are not [38–40] have been explored using the concept of epistemic injustice [41]. This term refers to the unfair treatment of some people in their capacity as knowing persons, and its relevance in the current context of acute concern with information and misinformation has been underlined [42]. Testimonial injustice, which is one tenet of epistemic injustice [41] occurs when someone is–typically inadvertently [43]–attributed a lower level of credibility due to belonging to a negatively stereotyped group. In healthcare, although the epistemic authority of caregivers is typically warranted due to their training and orientation toward scientific evidence [39], a care-seeking person may be subject to this type of injustice if their testimony is ignored, silenced or undervalued [38, 40]. Hermeneutic injustice, a second form of epistemic injustice [41], arises when a group of people struggles to understand their experience due to inadequate conceptual resources for doing so, for example due to limited availability of relevant research.

In the new digital informational landscape, where patients are able to take part of both other lay people’s experiences and medical research in ways not possible before, and where the patient-caregiver relationship has radically shifted towards enabling greater patient empowerment and contestation of medical authority [17, 18], a tension can be discerned between the importance of, on the one hand, countering misinformation and, on the other, maintaining democratization [15] of the ability to seek, discuss and question medical knowledge and practices, in line with the notion of epistemic justice [41]. Relatedly, tensions may arise in clinical meetings between caregivers and patients contesting established medical knowledge. This is of importance not least in to contraceptive counselling, where provider-patient interaction can strongly affect contraceptive choices [34, 44] and potentially create barriers to contraceptive use [27].

### The copper IUD

The importance of long-acting reversible contraceptive methods (LARC), including copper intrauterine devices (IUDs), for the prevention of unintended pregnancies is emphasized internationally and in Sweden [45]. The efficacy and safety of the copper IUD have been affirmed (e.g., [46, 47]) and Swedish medical guidelines point it out as suitable for most women who want to use a LARC method [48]. Established side effects include increased menstrual bleeding and pain on insertion [49]. The use of LARC has increased in Sweden, particularly among young women [50, 51]. In 2017, 6 percent of women aged 16–29 and 12 percent aged 30–44 were estimated to have used a copper IUD in the last year [52]. While research affirms user satisfaction with copper IUDs [53, 54], studies have also pointed to negative attitudes toward and experiences of the device, including in Sweden [35, 53, 55, 56]. In Sweden, where concerns about hormonal contraceptive methods have been noted [50, 52], a larger share of women have expressed concerns about using a copper IUD (52.3 percent) than a hormonal IUD (41.2 percent) [56]. A recent survey study [35] found that 34.7 percent of the respondents reported negative attitudes towards the copper IUD, while 45.4 percent of users indicated negative experiences, and open responses called for improved healthcare communication and updated research about the device. Apart from concerns with side effects, negative attitudes or experiences relate to, e.g., efficacy, device expulsion or unwillingness to insert a foreign object in the body [35] (cf. [53]).

Negative views on the copper IUD have often been associated with communication in social networks, particularly online, where negative commentaries on IUDs have been prevalent [11, 53, 57–59], although positive attitudes are also communicated [57, 59]. The importance of social networks for contraceptive choice and use has been observed, and accounts relayed through social contacts, or from individuals with personal experience, have been deemed by some to be more reliable than information from healthcare providers [11, 13, 25]. Studies observing negative attitudes toward the copper IUD and other contraceptive methods, not least as communicated online and through other social networks, have emphasized the importance of countering misinformation [11–13]. Meanwhile, researchers have expressed concern about caregivers approaching women’s experiences of or worries about side effects of contraceptives in terms of misinformation or myths [27, 32].

### Claims about systemic side effects of the copper IUD

Online communication about contraceptives has included claims about systemic side effects of the copper IUD, attributed to the release of copper from and chronic inflammation caused by the device. Noted symptoms are both somatic and psychological, and include anxiety, depression, panic attacks, fatigue, heart palpitations, weight gain, hair loss, skin problems, headaches and insomnia. Such reports have circulated internationally. They have been briefly noted in studies [56, 57, 60, 61] and addressed more in-depth in research from Sweden [35, 55, 62]. It may be mentioned that a large-scale safety and efficacy study of IUDs ([63], p 498) observed some cases of “symptoms compatible with hormonal side effects”, such as anxiety, depression, fatigue, weight gain, skin problems, dizziness and headaches, among copper IUD users. Another [49] found more severe side effects of copper IUDs in which the levels of copper were higher.

In Sweden, claims about systemic side effects of the copper IUD are largely centred on a social media group, founded in 2014 and now gathering around 8,700 members [55]. In a survey study about attitudes toward the copper IUD [35], 42 participants (2.1 percent) referred to copper toxicity or unestablished systemic side effects in response to open survey questions. While this is a small number, it is notable as this type of perceived or reported side effects was not mentioned in the survey or the accompanying text, thus suggesting that such notions are not entirely uncommon in Sweden.

Largely due to communication in the noted social media group, a number of suspected side effects of the copper IUD have been reported to the Swedish Medical Products Agency. This led to an investigation which concluded, in 2018, that no scientific evidence supports the claims [64]. Meanwhile, these claims have been referred to as an example of ‘alternative facts’ spreading through social media [65], expressive of broader tendencies to understanding claims contesting established medical knowledge in terms of misinformation [3].

### Healthcare principles and health-related claims

Evidence-based medicine (EBM), i.e., medical practice based on “integrating individual clinical expertise with the best available external clinical evidence from systematic research” [66], is a well-established principle according to which healthcare should be conducted, including in Sweden (e.g., [50]). EBM has been debated, for example with reference to tacit or individualized elements that do [67] or should [68] form part of medical practice. As noted by Mykhalovskiy & Weir [69], both medical and social scientists have pointed to risks involved in applying epidemiological knowledge about populations to the assessment of individuals (cf. [70]), through the standardization of clinical judgment and care. It has also been emphasized that EBM should encompass not only scientific research but also clinical experience and patient preference, and that EBM can thus be seen to align with the concept of ‘science and tried experience’ (*vetenskap och beprövad erfarenhet*) [71], on which healthcare in Sweden should be based [72]. Meanwhile, Miles and Loghlin [73] note that EBM is unable to incorporate patient perspectives when these are in conflict with the available science, arguing that EBM is therefore irreconcilable with person-centered care (cf. [74]).

Person-centered care (PCC) has become increasingly emphasized, in general (e.g., [73, 75]) and in contraceptive care (e.g., [76, 77]). In Sweden, following legal revisions emphasizing patient autonomy and involvement [78], healthcare–including contraceptive care [56] and other forms of healthcare for women [50]–should be person-centered [79]. While definitions of PCC vary [75, 80], it has been described as tailoring care to “to individual needs, preferences and circumstances by informing, engaging, and empowering patients” [80], or as “providing care that is respectful of, and responsive to, individual patient preferences, needs, and values and ensuring that patient values guide all clinical decisions” [81]. Noted aspects have included empathy, trust-building and an egalitarian patient-provider relationship [30], patient emotional well-being being prioritized, patient view-points being valued and respected [82], and focus being placed on the patient as a whole person [75]. As part of PCC, patients should be allowed to describe their ideas regarding their ailments, have their experiences taken seriously and their questions answered, and be given the opportunity to participate in decisions about their care [79]. In the context of contraceptive counselling, Holt et al. [77] emphasize that PCC entails prioritization of patient well-being rather than a more narrow focus on preventing unintended pregnancy at the potential expense of patient autonomy.

Alongside PCC, the maintenance of sexual and reproductive health and rights (SRHR) is an important principle for reproductive healthcare, internationally and in Sweden [50, 83]. The related concepts of reproductive justice and autonomy are also used in research (e.g., [28, 76]). The Swedish national strategy for SRHR emphasizes, for example, the right to acceptable methods of contraception and autonomous decision-making free from stigmatization or coercion. Healthcare should focus on the needs of the individual, all patients should be approached respectfully in ways promoting openness and trust, and all should have access to and be able to express the need for evidence-based information as well as relevant medical and social support, via physical and digital arenas [83]. The importance of caregivers adjusting care to individual patients’ needs, and of not communicating limiting norms, is also noted [50]. In the following, adhering to SRHR will be considered an aspect of PCC.

Regarding contraceptive care, a lack of knowledge on concerns about and experiences of LARC methods has been pointed out, despite the importance of understanding factors influencing contraceptive choices [24, 58], not least for the promotion of PCC and reproductive autonomy [32, 56, 76]. A paucity of research on how caregivers relate to patient concerns or dissatisfaction with contraceptives has also been noted [31, 32, 76], including when reported side effects have no (established) scientific basis [32]. This is despite the relevance of patient-provider interaction for contraceptive choice [34, 44] and use [27], as well as for PCC.

### Aim and research questions

This study aims to use the case of reported unestablished side effects of the copper IUD to investigate experiences of and reasoning about healthcare encounters between caregivers and patients contesting established medical knowledge. The following questions guide the study:

- How do women reporting that they have experienced systemic side effects of the copper IUD describe and reason about their encounters with healthcare?
- How do midwives and gynecologists reason about reports of such unestablished side effects and about how they do, would or should respond to them in the clinical encounter?

After an initial analysis of our material, a third question was formulated:

- How do the women’s and the caregivers’ concerns and reasoning relate to the healthcare principles of EMB and PCC?

## Methods and material

This is a qualitative study, based on seven online group interviews with, and 23 essays written by, members of the noted social media group centered on suspected side effects of the copper IUD, and on six online group interviews with midwives and gynecologists.

Participants from the social media group (SMG) (n = 35) were recruited through invitations posted in the group, with permission from the administrators. The group interview participants (= 23) were women aged 20–56 years, most with tertiary education, from across Sweden. Their professions included teaching, nursing, medicine, complementary medicine, research, and media communication. On the request of some SMG members who were reluctant to participate in an interview, an invitation to submit written essays on experiences of the copper IUD was also shared in the Facebook group. 23 essays, 1000–4800 words long, were collected, 11 of which were authored by interview participants. No sociodemographic data was gathered for the writers. All essay participants and 21 interview participants had personal experiences of health issues they associated with the IUD. The remaining ones had professional or scholarly interest in the issue.

Healthcare providers (HCP) (n = 15) were recruited through an advertisement in a national midwifery journal, an invitation posted on Facebook, and emails to reproductive healthcare clinics and professional networks. 12 were midwives and three gynaecologists, in line with 80 percent of contraceptive counselling in Sweden being conducted by registered midwives [84]. The participants were aged 30–70 years and had experience in working with contraceptive care as well as, for example, prenatal and maternal care, cervix-screening and abortion. They worked in clinics across Sweden. Three were in managerial positions, and one had been involved in the handling of contraceptives at the national level. All were women.

Essays were collected in October–December 2020, through an email message to LG. The group interviews were conducted via Zoom (2–4 persons/group), with SMG participants during March–December 2020 and with HCPs during January–June 2021, by both authors and with LG as principal convener. Our decision to conduct the interviews online, due to covid-19 and the geographical dispersal of participants, posed limits on the participant interaction associated with focus groups [85], which was our initial method choice. Therefore, and as two interviews had only two participants due to last minute cancellations, we term our method group interviews. Still, a significant degree of participant interaction occurred. The interviews were semi-structured, using an interview guide enabling the coverage of our main questions and of follow-up questions [86]. To ensure trustworthiness we asked probing questions about whether we had understood participants correctly [86].

Essay participants were instructed to describe (1) how they had come believe or suspect that copper IUD use may result in side effects unrecognized by healthcare, (2) the function of the Facebook group or other social media in this process, and (3) if they had been in touch with healthcare with regard to this issue and, if so, how they had experienced the healthcare encounter(s). The interviews with SGM participants were guided by the same questions. The HCP participants were asked about their experiences of and thoughts about reports of systemic side effects of the IUD, and about how such claims were and should be handled professionally in the clinic. The interviews were conducted in Swedish and lasted for 1–2.5 hours. After the noted number of group interviews, analytic saturation [87] was deemed to have been reached.

The interviews were audio recorded on a device separate from Zoom and transcribed verbatim. The anonymized transcripts and essays were analysed using a reflexive thematic approach [88, 89], aided by NVivo. Thematic analysis was chosen due to its theoretical flexibility and congeniality with our aim of identifying themes in the participant accounts [88]. Reflexive thematic analysis, which is characterised by, e.g., an emphasis on analysis as an active interpretive practice necessarily involving researcher subjectivity [89, 90], was deemed most suitable for our purposes. Accordingly, we did not bring pre-established codes or themes to the analysis, and we sought themes consisting of patterns of shared meaning underpinned by central organizing concepts, rather than content summaries [90, 91], in our material.

In line with Braun and Clarke’s [88] early delineation of thematic analysis, both authors first familiarised themselves with the data through close reading of the transcripts. Focused on the study’s first two research questions, MW worked through all transcripts inductively generating initial codes and sorting data segments into these based on their manifest [88] meaning. Searching for themes, she developed a focus on tensions related to principles of EBM and PCC, and the material was more deductively re-coded accordingly. LG and MW then reviewed, developed, defined and named the themes [88], in a collaborative iterative process, discussing and developing elements of the analysis and its grounding in the data (cf. [89]).

Adhering to a critical realist paradigm [92], we assume that reality exists with a degree of independence from conceptual frameworks used to understand it, and that some knowledge claims are more true than others [62, 93]. Thus, our material consists of participant accounts of more or less subjective strands of experiential, biological and medical knowledge which can and ought to be evaluated, not least scientifically. While such evaluation is of obvious value, and we have addressed aspects of this elsewhere [62], in this study we do not take a stance regarding the validity of participant claims about the copper IUD. Rather, we have striven towards a position of symmetry [94], i.e., of considering arguments from both participant groups as impartially as possible, which was communicated in the beginning of the interviews. This position was taken in line with striving towards epistemic justice [41], and with a feminist ethical imperative of taking research participants seriously [95]. Throughout the analysis, MW and LG engaged in reflexive dialogue about balancing between conflicting knowledge claims and their own assumptions about these (cf. [90, 91]).

MW is a public health and social work scholar and LG is specialized in gender studies. Both have experience in conducting interviews on sensitive topics. No previous relationships existed between authors and participants, except for one HCP who was personally acquainted with one author. Informed consent to participation was given at the beginning of the group interviews, and through the writing and submission of essays.

The study was approved by the Swedish Ethical Review Authority (2019–03017) and reported in line with Standards for Reporting Qualitative Research (SRQR) [90] (S1 Table).

## Results

SMG participants described of a range of somatic and psychological issues, such as those mentioned in the introduction, which they linked to copper IUD use. Their severity varied, having been seriously debilitating for some, in a few cases lasting up to 30 years during which the IUD was not connected to the symptoms and therefore not removed. While a few suspected the IUD to be the cause before coming across other people’s reports, most did not make that association until finding the social media group or similar forums. Alongside personal experiences [62], participants drew on scientifically oriented sources and arguments to found their claims and beliefs about the copper IUD [55]. Some reported a distinct weakening of symptoms after IUD removal, while others spoke of slower recovery or problems remaining.

Of the 15 HCP participants, 12 had heard of the social media group or the claims about copper-related side effects prior to being invited to this study. One had joined the social media group, due to an interest in learning about the issue. 12 had encountered reports of unestablished side effects in the clinic, for most only a few times. One participant referred to such reports as increasingly common. Another noted that patients may believe that they suffer from such side effects without mentioning it, for example when requesting IUD removal, “because… they know that we don’t agree with them” (HCP1:1). A few referred to a sense of stress in relation to the issue, due to feeling criticized by patients or pushed to take responsibility for something beyond their powers, and to pressure to avoid professional “failure” (HCG6:1) by ensuring the use of contraceptives, preferably LARC.

The analysis that follows is divided into four themes relating to the principles of EBM and PPC and the relation between these: *Principles of EBM*; *Principles of PCC*; *EBM vs*. *PCC*? and *Reconciling EBM and PCC* (Table 1).

**Table 1:** Themes and sub-themes.

*Themes*	*Sub-themes*
Principles of evidence-based medicine (EBM)	The imperative to follow clinical guidelines
	Problematizing the evidence-base of guidelines
	Reporting suspected side effects
Principles of person-centered care (PCC)	The obligation to listen to and respect the patient
	Experiences of limitations in PCC
	The potential relevance of gender
EBM vs. PCC?	Following guidelines vs respecting patients
	Lack of EBM and/or PCC: distrust
Reconciling EBM and PCC	Critically assessing and developing existing evidence
	Drawing on clinical and patient experiences
	Developing PCC

### Principles of evidence-based medicine

#### The imperative to follow clinical guidelines

Caregivers’ obligation to follow clinical guidelines, in line with EBM, was a central theme among the HCP participants. Discussing how reports or questions about unestablished IUD side effects should be met in healthcare, they agreed on the need to adhere to guidelines based on science:”we shouldn’t sit there and give advice that…. is not scientific” (HCP1:3).

We have a medical background and we do lean a lot on research and the evidence that exists and according to which we are obliged to work. (HCG5:2)

When asked, around half of the HCPs stated that they did not believe in the claims about copper-related side effects. They expressly said or appeared to tacitly assume that existing guidelines about the copper IUD have a firm evidence base, which made them skeptical of the claims. One participant stated that it has been”proven that … copper is not dangerous, not at all” (HCP1:2). Another noted that she trusted her midwifery training and that this is “the standpoint we should take” (HCP1:3). While the emphasizing of the scientific evidence-base of guidelines can thus be distinguished from more pragmatically observing the professional obligation to follow them, these two aspects seemed to fuse without friction for many HCP participants. As seen below, however, some expressed degrees of uncertainty.

#### Problematizing the evidence-base of guidelines

Several SMG participants expressed an understanding of the need and obligation of healthcare providers to follow clinical guidelines. For example,

I am not angry with the midwives and gynecologists that have, that I’ve seen, because they’ve just got, kind of, guidelines from above. (SMG1:1)

A central point of contestation here was, however, whether guidelines regarding the copper IUD are based in robust scientific evidence. SMG participants argued that these guidelines had not only failed to enable the alleviation of their health problems, but that they are also based on insufficient research. While some studies on links between IUD use and blood copper levels [96] were pointed out, gaps in such research were observed, as one aspect of a scientific orientation characterizing participants’ arguments [55].

[T]here is no scientific basis because no one has made any scientific studies. No, and that is why you don’t see any correlation. (SMG6:3)

This stated paucity of research was tied to regulatory demands being less rigorous for medical devices, including copper IUDs, than for pharmaceutical drugs (e.g. [97]). Relatedly, concerns with financial conflicts of interest potentially affecting both research and regulation were expressed. In brief, it was argued that while there is no scientific evidence of systemic side effects, nor does any scientific evidence disprove them [55].

Considering this, SMG participants argued that while following clinical guidelines, caregivers should be open to the problematization of their evidence-base. They pointed to the importance of critical thinking, including reflection on potential gaps and limitations in existing research. In the words of one participant, herself a medical doctor:

In healthcare we should work within, as it is called, science and proven experience. And the science is missing here. That’s what we need to open up for. (SMG1:2)

Around half of the HCP participants expressed some uncertainty regarding how to assess the reports of systemic side effects, with reference to medical science continually evolving and, in line with SMG participants, to a relative lack of research on the issue. Participants here pointed to”far too little synthesized information” (HCP3:1) and “a clear knowledge gap” (HCP5:2).

Yes, research is needed, quite simply. (HCP1:2)

One midwife remarked on not having sufficient knowledge to discern the truth value of the claims about copper-related side effects and having had trouble locating information about how to counter them. Some affirmed the importance of following clinical guidelines, underscoring their evidence-base, but later argued for taking the reports of side effects seriously and conducting more research on the matter. For example, the participant stating that it has been”proven that … copper is not dangerous, not at all” (HCP1:2), later referred to the importance of “listening to the women”, “taking them seriously”, and updating research on the matter.

We must follow the development–we cannot sit in the 70s and say that this copper IUD is good. “No, it’s not good”, they say… They you have to, kind of, look closer and deeper. (HCP1:2)

Meanwhile, one HCP participant argued that more research would likely not be seen as satisfactory by women reporting systemic side effects, as she perceived these claims to be driven by experiences of online community over and above sincere interest in medical research.

#### Reporting suspected side effects

As a basic way of monitoring post-marketing side effects signals [98], the reporting of suspected side effects is one tenet underpinning the development of EBM [68]. In line with this, SMG participants argued for the importance of reporting their suspected side effects and several had done so themselves. Many had also asked caregivers to do this, as reports coming from professionals were perceived to carry more weight. While some noted that caregivers had agreed, the accounts typically referred to reluctance. Some spoke of having asked more than one caregiver to make a report, with mixed results, and several described caregiver attitudes as dismissive. One participant mentioned having helped her caregiver make a report, after asserting the latter’s responsibility to do so. Others referred to having been told by caregivers to make reports themselves, in one case having been given incorrect information on how to go about it. Mixed messages about who bears the responsibility for reporting, and according to what rules, were also expressed. Participants noted having been told that midwives do not make such reports–”we don’t do that, they just said, we don’t do that” (SMG4:3)”–or that reporting the suspected side effects was not in accordance with existing guidelines. Moreover, an impression was expressed that reported side effects were not very closely followed up.

I have also reported those side effects (…) and I’ve contacted the midwife and asked her to make a report and, like this. But if feels like it’s a black hole. There’s no, kind of, nothing happens. (SMG 7:2)

One HCP participant noted having reported suspected unestablished side effects of the copper IUD. A few others remarked that they were unsure about how make such reports, overall or with reference to copper IUDs being medical devices rather than pharmaceutical drugs. Some stated that they and their colleagues were generally not very good at reporting side effects, due to lack of time or feeling it was not their responsibility. Two said they had encouraged patients to make reports, with one arguing that patients should describe their own symptoms.

[T]he message should come from the one who owns the experience, then it becomes real and truthful and us caregivers should not reformulate it. (HCP6:2)

A debated issue was which events that merit reporting. A few referred to pregnancy, uterine perforation or defective IUDs as proper occasions for reporting. A few affirmed that more unexpected or unestablished potential side effects should also be reported, while some argued that these do not warrant reporting as that requires belief in the plausibility of the causal link.

It’s also about us being licensed, that we should work according to, kind of, science and proven experience, and then that runs a bit counter to (…) report[ing] maybe a side effect that… that there is no scientific evidence for. (HCP1:1)

While some HCPs thus expressed reluctance to report potential side effects they did not believe in, SMG participants referred to a sense of a ‘catch 22’ situation whereby only proven side effects are judged to merit reporting, even though a function of such reporting is to identify previously unacknowledged effects. This suggests that existing evidence may constrain further development of EBM through processes shaping side effect signal reporting.

### Principles of person-centered care

#### The obligation to listen to and respect the patient

When HCP participants reflected on how to relate to patients referring to unestablished side effects, principles of PCC were commonly addressed. The imperative of listening to and respecting the patient, even when the caregiver does not agree with her, was highlighted here.

I do listen to what you say to me. I mean, I can’t dismiss what you say to me. All women are, like, unique. (HCP2:1)

After all, that’s our assignment, to meet and try to understand each other. (HCG5:2)

The importance of striving towards mutual understanding, and of the patient feeling satisfied with the healthcare visit, was noted. One participant emphasized that the health issue at hand is real for the patient, and that the caregiver must somehow meet her there.

I don’t believe in it, but at the same time (…) you should still show them respect, I feel. That they are feeling ill. (HCG1:1)

Aside from listening to patients being an obligation, some HCPs noted that this approach can decrease tension during the clinical encounter. Referring to a clinical meeting with a patient expressing suspicions about IUD side effects, one participant narrated:

I perceived her to be very relieved in that situation of not being questioned, to not get that reaction that, “no, do you know what dear, that’s not the way it is, because I know better” (…) I know it was a satisfied patient that left from there and that it’s largely about just that. That regardless of whether we think they are right or wrong, we have to listen and be humble to their experiences. (SMG3:2)

It was also stated, however, that such practices reflecting PCC principles were not always followed by caregivers in this and related contexts. The presence, at times, of a derogatory attitude or a “very negative tone” (HCP2:2) was noted:

Healthcare gives itself the right to declare people stupid [… which is] demeaning. (SMG2:2)

One participant mentioned having met patients expressing “guilt and shame” (HCP5:1), interpreting this as being due to previously not having been met with respect by caregivers.

They expect resistance, that I, as a healthcare provider, will kind of cut down on them and say “no, that’s not the case” or “no, that’s not correct”. (HCG5:1)

#### Experiences of limitations in PCC

In some contrast to HCP participants’ affirmation of the importance of listening respectfully to patients, a major theme in SMG participant accounts was not having felt respected or listened to by caregivers when seeking help. Participants referred to experiences of feeling belittled or offended, being laughed or sneered at, or declared stupid, crazy or hysterical by caregivers when expressing their suspicions about the IUD being the cause of their problems.

I was open about my suspected side effects in connection with removal. I was completely belittled by the doctor who all but scorned me and said I shouldn’t believe in things I read on Facebook. (Essay 2)

One participant mentioned not having been permitted to speak about her experiences:

[S]he interrupted me and then she said (…) yes but now it [i.e. the IUD] is out, now it’s good, now we move on. (SMG2:1)

Others referred to an unwillingness to discuss any potential explanation for the symptoms in question, once the copper IUD had been brought up.

[A]s soon as I had said I wanted to remove the copper IUD because I experienced side effects (…) he wasn’t interested in talking side effects. It’s not like he gave an alternative explanation (…) There was not even a conversation. It was just a, a stamp on my forehead and then full stop. (SMG3:1)

One woman described having been asked to leave the clinic:

Yes, she said if you continue to claim that you are poisoned by a copper IUD, there’s the door, you can go now. (SMG4:3)

Some emphasized that this had occurred when they were in a position of weakness due to illness, and that not having been taken seriously had added to their feeling of vulnerability:

[I]t’s been really hard to be sick but I almost think that’s been the worst, to not be believed, and to be so, yes, that you’ve felt like a child. (SMG4:1)

Many reported having been referred to mental healthcare, prescribed antidepressants or diagnosed with stress, despite having insisted that their ailments were somatic. It was remarked that once a mental health diagnosis is given, somatic care can become increasingly difficult to access. One participant did report having been taken quite seriously by her caregiver, commenting that this was possibly due to her being a medical doctor herself.

A discussion of direct relevance to SRHR [32, 99] concerned access to IUD removal. While many SMG participants reported having had their IUD removed on request, a few spoke of having faced resistance after disclosing their suspicions about side effects. Some reported having sought another caregiver, lied about the reason for requesting removal, or insisted on the right to make decisions about their body.

When I finally said “didn’t think I was going to be cross-examined when I’m the one who decides over my own body”. Then I was finally got help taking it out. (Essay 23)

Among HCPs, those addressing the issue agreed that patient requests for removal should be met. One mentioned having seen “desperate” (HCP4:2) patients who had sought but not been granted IUD removal by other caregivers. She added that while she would remove an IUD even if the patient had not used it for long, she would express her disapproval:

[I]f you don’t want it then I’ll take it out, but I still have to say I don’t think it’s what’s best for you! (HCP4:2)

Another HCP participant mentioned a degree of resistance toward IUD removal, with reference to the issue of needing to replace it with another contraceptive method.

#### The potential relevance of gender

In SMG participant discussions, issues related to gender and women’s health were brought up in relation to limited PCC, in all group interviews and some essays. Participants referred to a “deprioritization” (SMG3:2) of women’s health issues, which can be “swept under the carpet” (Essay 15) or “waved away” (SMG5:2), and historical examples expressive of a dismissive attitude towards women’s health issues were noted.

[T]here is still (…) some kind of, this nineteenth century view on women as hysterical (…) That you don’t take stories seriously (…) like no, but it’s just their imagination. (SMG7:3)

Similar issues were discussed among HCPs, although to a lesser degree. Some referred to women’s care being deprioritized, women not being taken seriously, and caregivers displaying condescending attitudes towards women. Meanwhile, some argued that this negative outlook on women’s healthcare should not be exaggerated. One stated that in comparison to other women’s health issues, the present one can be seen as less significant.

### Evidence-based medicine vs. person-centered care?

#### Following guidelines vs. respecting patients

The issue of how to navigate potential tensions between listening to the patient’s perspective and following clinical guidelines was central in the interviews with HCPs. After underscoring the need to follow clinical guidelines and research, one participant stated:

But this doesn’t mean that we shouldn’t respect someone’s experience and respect what she tells us (…) even though we don’t always believe that the women’s experience is what has really happened. (HCG5:2)

A distinction is made here between respecting “someone’s experience”, or “what she tells us”, and believing in her experience. Similarly, another participant put it concisely:

She is to be taken seriously, but the linking of the symptom to copper cannot be taken seriously. (HCP6:2)

While these accounts convey that it is possible to respect a patient’s subjective experience while dismissing her understanding of its objective causes, a sense of friction between adhering to EBM and listening to the patient was also noted.

[W]hen you talk about it, it almost feels contradictory, what we’ve said, kind of. That we are to meet [patients] with respect, and we are to listen, and so on, but at the same time… I mean I wouldn’t report this as a side effect (…) So, really, it feels a bit contradictory what I say. (HCP1:3)

Although not as explicitly spelled out, a corresponding contradiction can be traced in other HCP participants’ reflections. For instance,

You can’t neglect that they feel like this (…) but (…) nor can I, kind of, be swept away into something that I don’t… that maybe I don’t think is right. (HCP4:2)

Meanwhile, one HCP participant expressed the viewpoint that listening to and acknowledging a patient’s experience is difficult without affirming, at least to some extent, her understanding of that experience. She simultaneously pointed out that the social media group can be seen as a sign that healthcare has failed to meet and communicate constructively with some patients.

Somewhere healthcare has also failed in meeting these persons, which has caused them to have to seek in, for example, then, social media (…) Maybe we should meet these patients in this way so that they actually come to us and ask for advice if they have problems, so we can try to help them (…) To actually see the patient and say “yes absolutely, I hear you” and, I mean, that you validate the patient’s problems, that “it’s real. It’s not fake. You’re not stupid. You’re not crazy”. (HCP3:1)

Among the SMG participants, some also pointed to a sense of tension between caregivers’ obligation to adhere to EBM and their own wish to be treated in accordance with PCC.

Of course they have to balance a bit there. Both in relation to me, kind of, to understand me in my situation, and to be faithful to the grounds they have, which are (…) scientifically tested. (SMG2:4)

However, while principles of EBM and PCC were placed in a relationship of some opposition in HCP accounts, in SMG participant discussions they are not as easily distinguishable. Here they largely intertwined, as not having their understandings of the causes of their symptoms taken seriously was tied to not feeling respected as persons. For example, referring to wanting to be treated”as a thinking person” (SMG3:1), one participant noted that instead of just being dismissed, she would have preferred responses and discussions grounded in science:

[E]ven if these side effects had been imaginary, I would have liked to have been approached in a more correct and scientific way, with explanations. (SMG3:1)

Moreover, not having their beliefs about the causes of their symptoms taken seriously was linked not only to feeling disrespected, but to not being offered adequate help with symptom alleviation. When issues were attributed to mental rather than somatic factors, for example, this was not only perceived as offensive but also as an obstacle to diagnosis and cure. Thus, EBM was ineffectual.

It’s scary in that sense that we’re actually very (…) vulnerable because, I mean, I can’t go to healthcare and say, kind of, can you help me (…)

I feel completely left to myself (SMG5:1,3).

#### Lack of EBM and/or PCC: Distrust

Several SMG participants stated that their experiences of limitations in both EBM and PCC had caused their trust in healthcare to decrease. Concerning PCC, trust was noted to have diminished due to being “neglected” (SMG3:2) or badly treated.

You so trusted in healthcare, I did too. Now I only trust healthcare if I’ve broken a foot, kind of, other than that I don’t trust healthcare at all (…) because you’ve been so badly treated. (SMG4:3)

With regards to EBM, trust was noted to have decreased due to realizing, in the words of one participant, “how much we don’t know in medical science” (SMG1:2).

I guess I’ve seen conventional healthcare as very evidence-based (…) But in this case it doesn’t seem to be very evidence-based. So there I’ve, I’ve really had a change of views. (SMG3:1)

Noted consequences of this decrease in trust included refraining from seeking care for other ailments and seeking care from less evidence-based forms of practice, such as complementary or alternative medicine or experimenting on one’s own [55]:

[Y]es, he [the doctor] waved it away, kind of. Since then I’ve gone to alternative healthcare. (SMG5:2)

With aspects of EBM and PCC again intertwining, one participant stated that it should not come as a surprise that people become “questioning towards healthcare” when they are met with a bad “attitude towards us which is actually not based in very much research” (SMG3:2):

Then it’s healthcare itself that should work on the credibility capital that they’ve lost. (SMG3:2)

Both SMG and HCP participants pointed to the risk of growing distance and distrust between caregivers and patients. HCPs noted that decreased trust could spread to others who do not have similar experiences but come to expect them, as a “secondary distrust in healthcare” (HCP3:3), creating an “evil spiral when the trust in healthcare isn’t there” (HCP3:2). In sum, then, these accounts suggest that limitations in EBM, PCC or both can instigate and strengthen distrust in healthcare.

### Reconciliating evidence-based medicine and person-centered care

#### Critically assessing and developing existing evidence

Apart from indicating tensions related to, and between, EBM and PCC, the discussions suggested ways of resolving such tensions. A major argument made by SMG participants was that more research should be done on the safety of the IUD, to better undergird medical practice in line with EBM. Several HCPs also referred to a need for such research. In line with SMG participants, some of them stated that it impossible to say that the IUD does not cause any of the reported side effects, if no research exists to confirm that.

[T]hat’s why we need more research on the copper IUD (…) so it’s not myths but that we know more: this is the way it is or this is the way it is not. (HCP5:2)

In one case, further research was explicitly pointed out as a solution to the noted tension between following the principles of EBM and those of PCC.

[I]t almost feels contradictory, what we’ve said, kind of. That we are to meet [patients] with respect, and we are to listen, and so on, but at the same time (…) The solution would be (…) to do more research on it.

(…) I also think that would be the solution. That you did some scientific research. (HCG1:3,1)

Relatedly, many SMG and some HCP participants stated that caregivers should think critically about and be open to the fallibility and continuous evolution of medical science.

[Y]ou have to be able to question things (…) [J]ust because you question things it doesn’t mean that you’re unprofessional. (HCP2:2)

The notion that research should be conducted by “neutral” researchers (HCP2:2), in the sense of being unaffected by any vested interests, was also expressed.

#### Drawing on clinical and patient experiences

SMG participants argued that in absence of sufficient research, proven experience (*beprövad erfarenhet*) should be gathered through asking patients more routinely about experiences of contraceptive use and inquiring about contraceptives when investigating health issues.

[N]ow that the scientific evidence is missing (…) you have to work up the proven experience. And you do that by actually asking the patients; how did it go with the IUD, now, did you have any side effects, how has it worked? And work open-mindedly with that. (SMG1:2)

While asserting an understanding that clinical practice needs to rely on research, several SMG participants argued that caregivers ought to be able to mention to patients that some copper IUD users have experienced symptoms which they associated with the device:

[T]hat you, still, yes, there might be a possibility. We can’t confirm, but. (SMG7:1)

[I]t should be evidence-based and there must be science behind everything you prescribe and all that. But there’s actually nothing stopping anyone from saying that I have experience or I’ve heard from others that this has actually happened. (SMG1:3)

Correspondingly, one participant related that her midwife mentioning having heard stories of unestablished side effects of the IUD had led to removal and significant symptom alleviation.

SMG participants and some HCPs also argued for clarified and improved routines for following up patient experiences through reporting suspected side effects. SMG participants noted that this would improve both PCC and EBM, as taking individual patients seriously by making reports will enable any follow-up with potential implications for EBM.

The healthy way of reacting then should be no, but this doesn’t sound good. This sounds serious. Now let’s help each other report this so we can build a knowledge bank and, kind of, see that, OK, we are getting these amounts of reports, it’s time to do research on this (…) [Y]ou take it stepwise, sort of, through starting by taking each individual patient seriously. (SMG7:3)

It was pointed out, furthermore, that the social media group could be seen as an asset for healthcare, through signaling a potential need for, and offering input that might inform, research. This perspective contrasted with the views of some HCP participants, who spoke of health information circulating through social media as representing “fear-inducing campaigns” (HCP6:2) and as something of an antithesis to factual information.

#### Developing PCC

In addition, and as noted, SMG and HCP participants spoke of the value of caregivers treating patients respectfully. SMG participants expressed that they would have liked to have been listened to, and to have been shown “more empathy” (SMG3:2) and interest, in healthcare.

## Discussion

This qualitative study has used the case of reports of systemic side effects of the copper IUD, unrecognized by healthcare, to investigate experiences of and reasoning about encounters between caregivers and patients contesting established medical knowledge.

Both SMG and HCP participants pointed to tensions related to, and between, principles of EBM and PCC. Concerning EBM, a relative absence of research on potential side effects of the copper IUD was pointed out in all SMG groups and by some HCP participants, alongside a lack of clarity about routines for reporting suspected side effects. With regards to PCC, both participant groups observed the obligation of caregivers to listen respectfully to patients, while particularly SMG participants pointed to instances where this had not been seen in practice. A tension between caregivers’ obligations to adhere to EBM and to provide PCC was noted. While HCPs pointed to the possibility of listening to patients, in line with PCC, without taking the content of their claims seriously, among SMG participants these two aspects were connected, as not being taken seriously was tied not only to feeling dismissed but also to being inadequately helped. SMG participants and some HCPs pointed to potential resolutions of tensions related to PCC and EBM through caregivers assessing, developing and communicating relevant research, assembling proven experience, clarifying procedures for reporting and follow-up of suspected side effects and listening respectfully to patients.

### Mitigating lack of trust in healthcare: Developing EBM and PCC

Health-related communication online, including communication about suspected side effects of contraceptive methods, has in recent years increasingly been associated with the spread of misinformation, linked to distrust in healthcare [3, 5, 100]. In this study, SMG participants spoke about their own decreased levels of trust in healthcare, largely with reference to noted limitations in EBM and PCC. This is in line with a British survey study [101] connecting the erosion of trust in healthcare primarily to perceived limitations in professional expertise and PCC, the latter including not taking patients seriously, concluding that such aspects should be considered in efforts to increase public trust. Accordingly, while studies on attitudes towards IUDs recommend responding to negative accounts through countering misinformation [11, 13] and communicating positive stories of IUD use [11, 102], we suggest that healthcare providers’ efforts to mitigate lack of trust should also include identifying and working towards rectifying any existing tensions related to EBM and PCC.

### Identifying limitations in and developing PCC

It has been pointed out that diversion from PCC through dismissing patient concerns with potential contraceptive side effects, or resisting IUD removal, can contribute to resistance to IUD use [103]. Morison et al. [33] note that expert-directed decision-making in contraceptive care, in which patient knowledge or concerns are not considered, may lead to covert patient resistance through discontinuation of contraceptive use. Poor PCC in contraceptive care can also damage the patient-provider relationship [34, 104, 105] and increase distrust in healthcare [26, 106]. In other contexts too, such as among patients with contested diseases [107], distrust in medicine and withdrawal from care-seeking have been related to experiences of feeling deprioritized or badly treated by caregivers [6, 39, 40, 108]. Trust-building has meanwhile been conceptualized as an important part of PCC in contraceptive care [77], of relevance not least due to links between lack of trust in healthcare and resistance to contraceptive use [53].

Worth noting here, firstly, is that limitations in PCC [79] have been observed in research on contraceptive care, where caregivers have been noted to downplay, dismiss or neglect to discuss (concerns about) potential side effects of contraceptives including copper IUDs [24, 26–33, 35]. In a study of contraceptive care in Sweden [34], some women reported having felt patronized and bereft of agency, as their worries were dismissed and their experiences invalidated by caregivers. The researchers observed that due to the existing provider/patient power hierarchy, the affirmation of differing views or actions demanded strong self-efficacy on the part of the patient (cf [109]). In other studies, caregivers have been found to frame perceived contraceptive side effects in terms of problems with the patients themselves, as the patients’ own responsibility [27, 106], as excuses for IUD removal [30] or as fabricated [27, 32]. Relatedly, women with contested or medically unexplained disorders, such as chronic fatigue syndrome and fibromyalgia, have reported feeling dismissed and not being taken seriously by caregivers, as their illnesses were deemed imaginary or mental [107, 110]. These findings can all be related to accounts given by our SMG participants.

Some SMG participants reported having faced difficulties in getting their copper IUDs removed on request. While HCPs generally agreed that removal should be granted, some voiced a degree of reluctance. Although such reluctance is at odds with the principle of patient autonomy central to PCC, it is in line with previous research pointing patients who have met resistance when wanting to have their copper IUDs removed [26, 32, 35, 99, 104, 105, 111]. In correspondence with our results, previous research also indicates that some caregivers have leaned toward coercive practices to encourage LARC use [31, 106], including resisting LARC removal (e.g. [35, 99, 105]), through downplaying side effects, encouraging users to stick with the method despite concerns [28, 32], or requiring a valid reason or sufficient efforts toward compliance for removal [31, 99]. In a Swedish study some women reported that caregivers had not let them stop using their current contraceptive [34]. Such practices divert not only from principles of PCC but also of SRHR [26, 28, 32, 77, 105].

Secondly, previous research has indicated that caregivers agreeing with principles of PCC does not self-evidently guarantee that they practice accordingly. For one thing, clinical encounters in Sweden have been less patient-centered when patient concerns were multiple and not purely somatic [79]. Bodegård et al. [79] relate these findings to patients presenting complex or ambiguous medical problems tending to be perceived as “difficult” by doctors, suggesting that when challenged with medical uncertainty, caregivers may be less inclined towards PCC [79, 112, 113]. Moreover, healthcare providers have advocated PCC while simultaneously resisting IUD removal or sliding towards other forms of coercive LARC promotion [27, 30, 31]. Caregivers have thus described embracing PCC when considering requests for LARC removal, while in practice using tenets of PCC such as listening to patients, inquiring about and responding to their concerns, in strategic efforts aiming to delay or prevent removal [30]. Sidelining patient preferences and concerns, caregivers thereby used PCC to in effect undermine it [30]. Relatedly, one HCP participant noted that an open caregiver attitude is likely to increase patient willingness to consider other causes of symptoms than the claimed ones. While mutually finding strategies that fit the patient is a PCC priority (e.g., [56]), this actualizes the question of whether listening to the patient is seen as a way to bring her more into alignment with caregiver views, or as taking the patient’s viewpoint seriously. In sum, it is not entirely clear what PCC entails in meetings with patients whose health concerns do not align with the existing medical evidence.

Alongside limitations in PCC in contexts characterized by medical uncertainty [79, 107, 112–114], this, thirdly, brings the assessment of patient credibility to the discussion about PCC. Testimonial injustice is, as noted above, a tenet of epistemic injustice [41] occurring when someone is attributed less credibility due to belonging to a negatively stereotyped group. The person is thus wronged in her capacity as a provider of information, e.g. through acts of silencing or undervaluation [38]. In healthcare, although the epistemic authority of healthcare professionals is typically warranted due to their their scientifically oriented knowledge and training [39], a patient may be subject to this type of injustice if their testimony is ignored, or heard but not acknowledged as potentially relevant for the understanding of the medical situation, or if communication is closed down due to the caregiver being overly dismissive [40]. Pointing to the potential establishment of a climate of distrust in patient care, Buchman et al. [39] argue that to avoid epistemic injustice, caregivers should strive towards a disposition of epistemic humility. Clarifying that this does not mean rejecting clinical expertise or trusting all patients all the time, epistemic humility is described as willingness to engage in dialogue with the patient, including genuine inquiry into her experience, and critical reflection about assumptions made about her trustworthiness. This notion is congenial to wishes regarding healthcare encounters expressed by SMG participants. Their accounts align with all the noted forms of testimonial justice, with the dismissal of the potential medical relevance of their claims being one of their key concerns.

In light of the above, it is worth considering whether a climate of distrust [39] in women’s perceived side effects of contraceptives is present in clinical environments, and on whether caregivers’ efforts towards epistemic humility should involve reflection on how norms and expectations, including ones related to gender [41] (cf. [115]), might affect credibility judgments in the current context [39]. This is while SMG participants referred to breaches in PCC involving dismissal of their credibility (cf. [107, 114]). A clinical environment where it is not unusual for patient concerns with contraceptive side effects to be dismissed is not likely to strengthen trust in healthcare. We therefore join scholars arguing for training in and reflection on PCC in contraceptive care [28, 30, 104], with attention aimed towards epistemic humility [39] and cases characterized by medical uncertainty or complexity [79].

### Developing the evidence-base of copper IUD use

SMG participants, and some HCPs, argued that the evidence-base of copper IUD use should be strengthened, through more research and improved routines for reporting suspected side effects. Many related the paucity of research to weak regulatory demands for safety research on medical devices and some expressed concerns with financial conflicts of interests.

Any lack of relevant research is problematic for EBM, and for PCC in which provision of evidence-based information is an important part [50, 68, 77]. The Swedish national strategy for SRHR emphasizes, for example, that “everyone should know about, be able to express the need for, and have access to evidence-based information” [83]. This is in line with the inclusion of patient demands for well presented evidence in Greenhalgh et al.’s [68] delineation of real EBM. Accordingly, efforts of lay health movements in the contraceptive arena have often been focused on access to information about side effects [20, 115–117].

Weak regulatory demands for research on medical devices has been critiqued by healthcare professionals (e.g., [97, 118]), with new legal requirements gradually being put in place in the EU since 2017 [119]. Regarding the reporting of suspected side effects, research from Sweden corroborates participant accounts of underreporting and uncertainty about what and how to report [120], despite the importance of such reporting for post-marketing research and EBM [68, 120], and medical product withdrawals due to safety problems often being based on data from such systems [121]. Notably, some of our HCP participants related making judgments based on established research about what perceived side effects that merit reporting, which may constrain further development of scientific inquiry and EBM.

Concerns about financial conflicts of interests have also been voiced in research. Greenhalgh et al. (2014) argue that the main challenge to EBM is hidden biases due to vested interests of pharmaceutical and medical device industries, and researchers have affirmed that heightened efforts to curb conflicts of interests in medical research would not only benefit EBM but decrease mistrust in healthcare [39] and the spreading of misinformation [6, 122, 123]. Moreover, historical evidence of past examples of negative effects of contraceptives and other reproductive health devices and their methods of distribution, which have initially gone unrecognized [20, 21, 23, 118], may affect perceptions of the trustworthiness of [124], and contribute to an attitude of epistemic humility on the part of, healthcare providers.

Thus, while a few HCPs noted that more research or improved reporting routines would likely not be accepted as sufficient by patients claiming unestablished side effects, we deem it reasonable to assume that stronger regulatory demands for research and clarified procedures for reporting suspected side effects would make healthcare less open to criticism and distrust, particularly when considering the scientific orientation of many of the SMG participant’s arguments [55]. This is while Buchman et al. (2017) note that trust in providers, and the latter’s privileged position in the epistemic hierarchy, are largely based on their science-based knowledge. Relatedly, while writing about the perils of misinformation on contraceptives, Foran [12] argues that providing evidence-based information should be top priority. Worth adding here is that developing research, in areas where science may hitherto be undone [115, 125, 126], is important for discerning what can be defined as evidence or as misinformation.

The paucity of research can be seen as a claim to hermeneutic injustice [41], the form of epistemic injustice which arises when a group of people faces trouble when trying to understand their experience due to inadequate conceptual resources, resulting in their marginalization. While the lack of interpretative resources results in collective inadequacy of understanding of the phenomenon, the disadvantaged group is prevented from understanding their own experience [41]. This is one possible interpretation of the situation of the women claiming unestablished side effects of the copper IUD.

### Straddling EBM and PCC: Considering patient experiences

Among HCP participants, further research was pointed out as a potential resolution to the sometimes explicitly noted tension between PCC and EBM, i.e., between listening to the patient and taking her claims seriously. The reporting of suspected side effects was also noted as a means to resolving this tension. As one SMG participant put it, the reporting of possible side effects can be a way of strengthening PCC by taking the patient seriously, while also enabling the development of EBM through the evaluation of any build-up of similar reports.

Moreover, the tension between principles of EBM and PCC noted in the participant accounts can be related to observations of a general conflict between EBM and PCC, i.e. between following standardized guidelines based on scientific population-level study and tending to individual patient needs [68–70, 73]. While Downey et al. [74] refer to discussions of EBM highlighting tensions between scientific data and patient experiences, Miles and Loghlin [73] observe, as noted above, that an inability of EBM to incorporate patient perspectives when these conflict with the available science indicates an incommensurability between EBM and PCC. Attempts to overcome this tension have been conceptualized in terms of evidence-informed individual care [73], patient-based evidence [127] or real EBM emphasizing the clinician-patient relationship and the care of the individual [68].

Researchers have also argued that dominant paradigms in contraceptive care may rely on a narrow definition of scientific evidence, and that in order to be appropriate and patient-centered it should also engage with evidence grounded in patients’ experiential knowledge [33, 74]. Invoking reproductive justice, Fulcher et al. [128] state that contraceptive counselling should acknowledge the importance of experiential information, and include discussion of accounts patients may have received from different sources [128]. It has been noted that while many patients, across racialized and class groups [27], prioritize embodied experience in contraceptive decision-making [25], although it is often combined with available biomedical knowledge [55], the reliance on epistemically privileged biomedical knowledge enables the overlooking of patient experiences, including the understanding of perceived side effects in terms of fabrications or misconceptions [27, 32, 106]. This, Berndt and Bell argue [27], can entail a major interactional barrier to contraceptive use.

This sense of conflict between forms of evidence or knowledge can be juxtaposed with studies pointing to lay perceptions of caregivers as a source of positively charged information about the copper IUD, whereas negative accounts, and information about side effects, are primarily shared via social contacts [11, 35, 44]. While negative accounts may be regarded as more memorable than positive ones, and attract more attention [5, 11, 12], this sense of discrepancy [35] is noteworthy not least due to conflicting messages being conducive to lack of trust in healthcare [11] and the spread of misinformation [5].

While a few SMG and HCP participants noted that the social media group in question could be viewed as a resource for healthcare, signaling a need for and potentially contributing to medical research, Richards et al. [129, p 7] argue that online patient communities can be seen as “a rich and as yet largely untapped learning resource for health professionals”. Patient-driven efforts have contributed to medical knowledge, for example through evidence-based activism [130] and pointing to gaps in scientific knowledge [125], including in cases where contested illnesses have progressively become considered as real medical issues [131]. This poses questions about conditions under which patient perspectives can and should be considered or integrated into clinical communication or research, and about how trust in caregiver expertise should be balanced against trust in the experiences of patients [39].

### Limitations

The SMG participants were highly educated overall, and likely more engaged and well-resourced than many other members of the social media group. In turn, the HCPs were probably more interested in the topic of potential systemic side effects than many other caregivers. These issues are, however, common to many qualitative studies, and do not detract from the relevance of the themes generated in our analysis.

While the accuracy of our interpretations were not checked by participants [132], the trustworthiness of our results were ensured through probing questions during the group interviews and through the authors’ collaborative analysis encompassing meticulous discussion of the analytic themes and their grounding in the empirical data, including reflexive dialogue about balancing between participant claims and the authors’ own assumptions about these.

While the generalizability of elements of our analysis to other cases of clinical communication involving lay contestation of established medical knowledge may be limited, we believe that our arguments can be used in reflection on and assessment of other such cases, not least in so far as, as noted above, our findings echo, and add new nuances to, previous findings concerning contraceptive care.

## Conclusions

This study of how women making claims about systemic side effects of the copper IUD reason about their encounters with healthcare, and how caregivers reason about how such claims are or should be met, points to tensions related to the principles of EBM and PCC. Thus, while studies on attitudes towards IUDs recommend responding to negative accounts through countering misinformation [11, 13] and communicating positive stories of IUD use [11, 102], we suggest that efforts to counteract lack of trust in healthcare should also include the identification and rectification of any tensions related to EBM and PCC, by ensuring and communicating adequate research, clarifying procedures for reporting and following up suspected side effects, and listening respectfully to patients [39] in a way that does not exclude acknowledgment that patient understandings can be of potential medical relevance. This can increase the quality of care while contributing to mitigation of distrust in healthcare.

## Supporting information

S1 Table Standards for Reporting Qualitative Research (SRQR) checklist. The table shows the Standards for Reporting Qualitative Research (SRQR) checklist, with status marked X indicating that the study follows the respective recommendation in the way and to the degree deemed appropriate by the authors.
(DOCX)
