# Supplementary material for: Claims in the clinic: A qualitative group interview study on healthcare communication about unestablished side effects of the copper IUD
Source: PLoS One. 2023 Sep 28;18(9):e0291966. doi: 10.1371/journal.pone.0291966 (PMC10538671; doi:10.1371/journal.pone.0291966)
Supplement: S1 Table — The table shows the Standards for Reporting Qualitative Research (SRQR) checklist, with status marked X indicating that the study follows the respective recommendation in the way and to the degree deemed appropriate by the authors. (DOCX) [file pone.0291966.s001.docx]

**S1 Table: Standards for Reporting Qualitative Research (SRQR) checklist**

The table shows the Standards for Reporting Qualitative Research (SRQR) checklist (1), with status marked X indicating that the study follows the respective recommendation in the way and to the degree deemed appropriate by the authors.^1^

| No | Topic | Item | Status |
| --- | --- | --- | --- |
|  | **Title and abstract** |  |  |
| 1 | Title | Concise description of the nature and topic of the study identifying the study as qualitative or indicating the approach (e.g., ethnography, grounded theory) or data collection methods (e.g., interview, focus group) is recommended. | X |
| 2 | Abstract | Summary of key elements of the study using the abstract format of the intended publication; typically includes background, purpose, methods, results, and conclusions. | X |
|  | **Introduction** |  |  |
| 3 | Problem formulation | Description and significance of the problem/phenomenon studied; review of relevant theory and empirical work; problem statement | X |
| 4 | Purpose or research question | Purpose of the study and specific objectives or questions. | X |
|  | **Methods** |  |  |
| 5 | Qualitative approach and research paradigm | Qualitative approach (e.g., ethnography, grounded theory, case study, phenomenology, narrative research) and guiding theory of appropriate; identifying the research paradigm (e.g., postpositivist, constructivist/interpretivist) is also recommended; rationale. | X |
| 6 | Researcher characteristics and reflexivity | Researchers’ characteristics that may influence the research, including personal attributes, qualifications/experience, relationship with participants, assumptions, and/or presuppositions; potential or actual interaction between researchers’ characteristics and the research questions, approach, methods, results, and/or transferability | X |
| 7 | Context | Setting/site and salient contextual factors; rationale | X |
| 8 | Sampling strategy | How and why research participants, documents, or events were selected; criteria for deciding when no further sampling was necessary (e.g., sampling saturation); rationale | X |
| 9 | Ethical issues pertaining to human subjects | Documentation of approval by an appropriate ethics review board and participant consent, or explanation of lack thereof; other confidentiality and data security issues | X |
| 10 | Data collection methods | Types of data collected; details of data collection procedures including (as appropriate) start and stop dates of data collection and analysis, iterative process, triangulation of sources/methods, and modification of procedures in response to evolving study findings; rationale | X |
| 11 | Data collection instruments and technologies | Description of instruments (e.g., interview guides, questionnaires) and devices (e.g., audio recorders) used for data collection; if/how the instrument(s) changed over the course of the study | X |
| 12 | Units of study | Number and relevant characteristics of participants, documents, or events included in the study; level of participation (could be reported in results) | X |
| 13 | Data processing | Methods for processing data prior to and during analysis, including transcription, data entry, data management and security, verification of data integrity, data coding, and anonymization/deidentification of excerpts | X |
| 14 | Data analysis | Process by which inferences, themes, etc., were identified and developed, including the researchers involved in data analysis; usually references a specific paradigm or approach; rationale | X |
| 15 | Techniques to ensure trustworthiness | Techniques to enhance trustworthiness and credibility of data analysis (e.g., member checking, audit trail, triangulation); rationale | X |
|  | **Results/findings** |  |  |
| 16 | Synthesis and interpretation | Main findings (e.g., interpretations, inferences, and themes); might include development of a theory or model, or integration with prior research or theory | X |
| 17 | Links to empirical data | Evidence (e.g., quotes, field notes, text excerpts, photographs) to substantiate analytic findings | X |
|  | **Discussion** |  |  |
| 18 | Integration with prior work, implications, transferability and contribution(s) to the field | Short summary of main findings; explanation of how findings and conclusions connect to, support, elaborate on, or challenge conclusions of earlier scholarship; discussion of application/generalizability; identification of unique contribution(s) to scholarship in a discipline or field | X |
| 19 | Limitations | Trustworthiness and limitations of findings | X |
|  | **Other** |  |  |
| 20 | Conflicts of interest | Potential sources of influence or perceived influence on study conduct and conclusions; how these were managed | X |
| 21 | Funding | Sources of funding and other support; role of funders in data collection, interpretation, and reporting | X |

^1^In line with recommendations from Braun and Clarke (2), we here note their argument that the quality and trustworthiness of reflexive thematic analyses, which is the method used in this study, is not most suitably ensured by following checklists for qualitative studies. This is as such checklists tend to be built on a “procedural rather than philosophical definition of qualitative research, and values and quality standards that often reflect neo-positivist and (naïve) realist assumptions” (2, p 13). They emphasize that the quality of reflexive thematic analysis is strengthened through theoretical and reflexive deliberation and explicit decision-making, while no analysis can be entirely “accurate” as it consists of an inherently interpretive practice and meaning is not statically and entirely objectively located in in empirical data.

**References**

1. O’Brien B, Harris IB, Beckman TJ, Reed DA, Cook DA. Standards for Reporting Qualitative Research: A Synthesis of Recommendations. Acad Med 2014;89(9):1245-51.

2. Braun V, Clarke V. Is thematic analysis used well in health psychology? A critical review of published research, with recommendations for quality practice and reporting. Health Psychol Rev. 2023:1-24.
